# Electrophysiological indices of reward anticipation as ADHD risk and prognostic biomarkers

**DOI:** 10.1007/s00787-024-02606-4

**Published:** 2024-11-08

**Authors:** Nóra Bunford, Kristóf Ágrez, György Hámori, Júlia Koller, Attila Pulay, Zsófia Nemoda, János M. Réthelyi

**Affiliations:** 1 Clinical and Developmental Neuropsychology Research Group, HUN-REN Research Centre for Natural Sciences, Budapest, Hungary; 2 Department of Cognitive Science, Budapest University of Technology and Economics, Budapest, Hungary; 3 Institute of Genomic Medicine and Rare Disorders, Semmelweis University, Budapest, Hungary; 4 Department of Psychiatry and Psychotherapy, Semmelweis University, Budapest, Hungary; 5 Department of Molecular Biology, Semmelweis University, Budapest, Hungary

**Keywords:** ADHD, Biomarker, Event-related potential, Polygenic risk score, Adolescent

## Abstract

The attention-deficit/hyperactivity disorder (ADHD) clinical phenotype has limitations for deciphering ADHD etiology and predicting prognosis. Although relative to the clinical phenotype, intermediate phenotypes may have better explanatory and prognostic power, the extent to which ADHD intermediate phenotypes are associated with ADHD risk and prognosis is unknown. The aim of this study was to evaluate evidence for event-related potential (ERP) measures of reward anticipation as ADHD risk and prognostic biomarkers. We examined, whether (1) in a sample of adolescents (*N* = 304;* M*_age_ = 15.78 years, *SD* = 1.08; 39.5% female), accounting for the effects of age, sex, ADHD severity and depression, ERPs are associated with ADHD polygenic risk scores (PRSs) (ADHD risk) and (2) in a sample of adolescents at-risk for ADHD (*n* = 99; *M*_age_ = 15.78 years, *SD* = 1.08; 39.5% female), accounting for the effects of age, sex, ADHD severity, depression, and baseline outcome values, ERPs are associated, prospectively, with alcohol misuse (ADHD prognosis). In adolescents, greater ADHD PRSs were associated with lower electrophysiological anticipatory attention to motivationally relevant feedback (*b* = −0.115, *p* = .046 95%CI [−0.228; −0.002]). The predictors accounted for 5% of the variance in the outcome. In adolescents at-risk for ADHD, at 18-month follow-up, lower electrophysiological anticipatory attention to motivationally relevant feedback was associated with greater alcohol consumption (*b* = −7.454, *p* = .007, 95%CI [−12.873; −2.034]). The predictors accounted for 31% of the variance in this outcome. These findings were replicated in sensitivity analyses with behavioral performance variables added as covariates. The current findings support the hypothesis that ERP amplitudes of reward anticipation may be ADHD risk and prognostic biomarkers and suggest that intermediate phenotypes may confer advantages over the ADHD clinical phenotype in delineating etiology and predicting prognosis.

**Supplementary Information:**

The online version contains supplementary material available at 10.1007/s00787-024-02606-4.

## Introduction

Attention-deficit/hyperactivity disorder (ADHD) is an early-onset, functionally impairing and prevalent disorder that is associated with greater risk for a host of negative and impairing outcomes [1], including alcohol problems [2]. Adolescents and adults with ADHD are at greater risk for developing alcohol use disorders and problems, and in individuals with ADHD, the lifetime prevalence of alcohol dependence is ~ 3–11% and of any alcohol use disorder, it is ~ 43% [2]. The comorbidity of ADHD with alcohol use disorder is associated with additional comorbidities and worse response to treatment [2]. Adolescent alcohol use is associated with attenuated grey matter and deficits in cognitive processes affected by ADHD, including attentional and executive functions, leading to worse cognitive and developmental outcomes for adolescents with ADHD who frequently consume alcohol. In turn, worse adolescent cognitive outcomes are associated with greater risk for adulthood alcohol binge drinking and, over time, worse health and socioeconomic status [2]. Better understanding the causes of ADHD and identifying predictors in adolescence of prognosis in young adulthood is key to advancing the effectiveness of early identification of at-risk individuals and to the individualization of prevention and treatment, i.e. precision psychiatry.

A clinical phenotype is the clinically observable and relevant characteristics of a disorder, i.e. manifest symptoms. Data indicate efforts to determine etiology and predict prognosis relying on the clinical phenotype have been largely unsuccessful, arguably because the ADHD clinical phenotype is heterogeneous in terms of causes, manifestation, and course [3, 4]. Specifically, a multitude of developmental pathways can lead to a clinical phenotype that is multifaceted both with regard to core symptoms (i.e., difficulties with regulating activity, attention, and impulses [1]), and with regard to associated features (e.g. emotional features [5], executive functioning [6], and reward processing [7]). This multifaceted clinical phenotype, in turn, leads to diverse outcomes.

Intermediate phenotypes are biological markers that are heritable and, considering an etiological framework, located between genetic predisposition and manifest symptoms [8, 9]. Relative to the clinical phenotype, intermediate phenotypes, by virtue of their homogeneity, are hypothesized to have better explanatory and prognostic power [7]. Evidence indicates reward processing may be an ADHD intermediate phenotype, as findings show associations between differences in reward processing and ADHD [3, 4, 7]. Research on biomarkers of reward processing in ADHD is comprised almost exclusively of case–control and diagnostic biomarker studies. A *diagnostic biomarker* (1) confirms or detects the absence/presence of a condition and/or (2) differentiates across presentations (subtypes) of that disorder [10]. In many cases, between-group differences across ADHD and control groups were not detectable/ replicated [11–14], or biomarkers did not differentiate diagnostic groups [7], leading to the conclusion that the biomarker is clinically irrelevant or uninformative. Yet, findings of case–control studies may be misleading as even in the absence of between-group differences in the biomarker, there may be a difference in the extent to which (or whether) the biomarker is associated with functional outcomes. In case of reward processing, even in the absence of between-group differences in neural reward response, there is a between-groups difference in how neural reward response is associated with affective and alcohol outcomes. For example, in adolescents at-risk for ADHD, a negative association was observed between neural reward response and depression and a positive association was observed between neural reward response and hazardous alcohol use. In adolescents not at-risk for ADHD, neural reward response was not associated with depression and it was negatively associated with hazardous alcohol use [14]. By definition, diagnostic biomarker studies assess the extent to which a given biomarker of an intermediate phenotype converges with the categorical clinical phenotype even though the very utility of the biomarker lies in being an improvement upon and thus nonredundant with the clinical phenotype. Both case–control and diagnostic biomarker studies, albeit informative about differences at the group level, are by nature uninformative about causes and course.

Taken together, the test of clinical utility of a biomarker of an intermediate phenotype is not whether it differs or differentiates between groups, i.e. whether it is a diagnostic biomarker. Rather, the apt test of such utility is whether it explains the causes of or the course of the disorder, i.e. whether it is a risk or a prognostic biomarker [7]. A biomarker that indicates the potential for developing a disorder or medical condition in an individual who does not currently have clinically apparent disorder or the medical condition is classified as a *susceptibility/risk* biomarker. The concept is similar to prognostic biomarkers, except that the key issue is the association with the development of a disease rather than prognosis after one already has the diagnosis [10]. *A prognostic biomarker* indicates the likelihood of a clinical event or outcome, or the progression or recurrence of the disorder in individuals with the condition [10].

Event-related potentials (ERPs) are changes in the electroencephalogram (EEG) as a result of specific events (i.e. stimuli) that reflect, physiologically, the synchronous activity of neuronal populations and psychologically, different cognitive functions, e.g. affective, cognitive, motor, of perceptual processes that are experimentally probed by stimuli or a task [15]. ERPs are appropriate and ideal for assessing aspects of reward processing defined and differentiated based on their occurrence in time [16]. Moreover, given their acceptance by participants, cost effectiveness, and relatively high movement tolerance, ERPs are also suitable for collecting data from large clinical samples longitudinally [17, 18]. Case–control studies indicate between-group differences in ERPs to reward across ADHD and control groups, e.g. adolescents and children with ADHD exhibited enhanced ERPs to escaping delay [19] and to salience of reward [20] as well as greater improvements in behavioral inhibition as a result of social rewards [21].

In some cases, between-group differences in ERPs to reward across ADHD and control groups were not detected, e.g. between adults and children with and without ADHD to error and to inhibition [22–24], to probabilistic reward learning [25], or with regard to improved performance as a result of reward [26]. Diagnostic biomarker studies indicate in adolescents, ERPs of reward do not differentiate adolescents with and without ADHD [7].

Here, we examine whether electrophysiological indices of reward processing are ADHD risk and prognostic biomarkers. We index ADHD risk by ADHD polygenic risk scores (PRS), which reflect the cumulative effect of frequent genetic variants [5], and index ADHD prognosis via alcohol use. Specifically, our aims were to examine whether (Aim 1) in a sample of adolescents, ERP measures of reward anticipation are associated with ADHD PRSs, and whether (Aim 2) in a sample of adolescents with the ADHD clinical phenotype, ERPs of reward anticipation are associated, longitudinally, with alcohol use. We hypothesized that ERP measures of reward anticipation are associated with ADHD PRSs and longitudinally, with alcohol use.

Across analyses, we account for the effects of age, sex and depression, given an established link between reward processing and these variables [27, 28]. We also account for the effects of ADHD severity; first, to ensure that its shared variance with ADHD PRSs does not account for findings and second, to ensure that any findings obtained reflect effects of the intermediate phenotype beyond the clinical phenotype.

### Methods

#### General procedure

Data analyzed in the current study were collected at the first two assessment sessions of the second (Wave 1) and fourth (18-month follow-up, i.e. Wave 2) years of a longitudinal study, the Budapest Longitudinal Study of ADHD and Externalizing Disorders.

Participants were excluded if they exhibited cognitive ability at or below the percentile rank that corresponds to a full-scale IQ score of 80 on the Wechsler intelligence scale for children–Fourth Edition (WISC-IV) or the Wechsler adult intelligence scale–Fourth Edition (WAIS–IV) [29, 30]; met diagnostic criteria for bipolar, obsessive–compulsive or psychotic disorder on the Structured Clinical Interview for DSM-5 Disorders, Clinical Version (SCID-5-CV); had a prior autism spectrum disorder (severity ≥ 2) diagnosis; reported a neurological illness; and had visual impairment (uncorrected, impaired vision < 50 cm).

Following written informed assent (adolescents) and written informed assent (parents), adolescents completed a series of tests. At Wave 1, the first assessment session comprised clinical interview and cognitive testing, genetic sampling, and completion of questionnaires. The second assessment session comprised an EEG measurement and completion of questionnaires. At Wave 2, the first assessment session comprised completion of questionnaires. The second assessment session comprised an EEG measurement. Questionnaires were completed by parents via Psytoolkit [31, 32] and Qualtrics (Version June 2020–May 2023) (Qualtrics, Provo, UT). The longitudinal study was approved by the National Institute of Pharmacy and Nutrition (OGYÉI/17089-8/2019). The study has been performed in adherence to the ethical standards of the 1964 Declaration of Helsinki and its later amendments.

ADHD classification was determined using parent-report on the ADHD Rating Scale-5 (ARS-5) [33]. To be classified as at-risk for ADHD, adolescents had to meet a total of ≥ 4 of the Diagnostic and Statistical Manual of Mental Disorders (5th ed.; DSM-5) ADHD symptoms (from either domain). To be classified as diagnosed with ADHD (for purposes of research), adolescents had to meet a total of ≥ 6 (youth < 17 years old) or 5 (youth ≥ 17 years old) of the DSM-5 ADHD inattentive (IA) or hyperactive/impulsive (H/I) symptoms and exhibit impairment (i.e., rating of ≥ 2 = moderate impairment) in ≥ 3 areas of functioning.

Addressing different questions, findings with samples drawn from the larger longitudinal study, have been previously published [7, 14, 34–36].

### Participants

Participants were *N* = 304 adolescents oversampled for ADHD, i.e. recruited from the community and hospitals, as detailed in previous publications [34, 35]; at baseline, adolescents were between 14 and 17 years (*M*_age_ = 15.78 years, *SD* = 1.08; 39.5% female); *n* = 132 (43.4%) met criteria for at-risk for ADHD. At Wave 2, data were available for *n* = 233 adolescents (23% attrition), of whom *n* = 99 (42.5%) were classified at baseline as at-risk for ADHD. At Wave 2, adolescents at-risk for ADHD were between 15 and 19 years (*M*_age_ = 17.08 years, *SD* = 1.07; 29.3% female).

Participants’ average cognitive ability was in the 61st percentile (*SD* = 20.86). Based on net household income per person, compared to the 2020 Hungarian average of ~ 147 000 HUF [37], with a sample average of 156 374 HUF (*SD* = 77 685), participating adolescents and their families were from a somewhat above-average socioeconomic background *t*(303) = 2.104, *p* = 0.036, Cohen's *d* = 0.121 (95% CI[0.008, 0.233]). For details on medication washout, see Supplement.

### Measures

#### Rating scale measures

Items from the self-reported European School Survey Project on Alcohol and Other Drugs (ESPAD) master questionnaire [38] were used to assess alcohol consumption, binge drinking, and drunkenness across lifetime, during the last 12 months, and during the last 30 days. The parent-reported ARS 5 [33] was used to assess ADHD. Prior findings indicate acceptable psychometric properties for both the ESPAD [34, 38–41] and the ARS 5 [7, 14, 33]. In the current sample, the Binge Drinking (ω_baseline_ = 0.940; ω_T2_ = 0.938), the Consumption (ω_baseline_ = 0.916; ω_T2_ = 0.934), and the Drunkenness (ω_baseline_ = 0.944; ω_T2_ = 0.938) subscales of the ESPAD and the ARS-5 Total (ω_baseline_ = 0.954) exhibited acceptable internal consistency and were used in analyses. For details, see Supplement.

### Monetary incentive delay (MID) task

The MID task [42, 43] is the recommended task for probing reward anticipation [44] and its electrophysiological version, the e-MID task is appropriate for differentiating electrophysiological response to anticipation and receipt of reward [45]. Evidence indicates reliability of e-MID ERPs [16] as well as convergent validity between e-MID ERPs and self-report reward processing [46]. For description of the employed MID parameters and version, see Supplement. For analyzed ERP variables, see *Analytic Plan*.

### EEG data acquisition and processing

Details and procedures for EEG data recording and processing have been described previously [7]. Electrodes and time windows were selected based on the literature [7, 16, 27, 45, 47] based on when and where ERPs were maximal during our pilot studies: Cue P3 at Pz, POz, P1, and P2, for the 450–650 ms time window; Target P3 at CPz, Pz, P1, and P2, for the 200–375 ms time window; SPN at CPz, Pz, CP1, CP2, P1, and P2, for the -200–0 ms time window; and RewP at CPz, Cz, FCz, CP1, CP2, FC1, and FC2, for the 225–325 ms time window [7].

### Genotyping

Genomic DNA was isolated from saliva samples. Samples were processed following manufacturer guidelines and recommendations [48] and genotyped using the Illumina Infinium Global Screening Array-24 v3.0 BeadChip by LIFE & BRAIN GmbH (Bonn, Germany).

### Analytic plan

All analyses were conducted in RStudio (version 2023.09.1. Build 494, R version 4.3.2.). For packages used, see Table S1.

Data preparation involved imputation of missing data. Missing alcohol use data were substituted using multiple imputation with a state-of-the-art deep learning method, for details see [49]. One of five generated datasets was used.

### PRS

ADHD PRSs were calculated based on a discovery dataset involving 38,691 individuals with ADHD and 186,843 controls [50]. Using SNP cutoff of *p* < 0.50, the number of ADHD PRS SNPs was 99,330 with an associated *R*^2^ of ≈3.7%. For details, see Supplement.

### Statistical analyses

*Exploratory factor analysis (EFA)* EFA was conducted with the aim of dimension reduction, on 48 ERP variables: indices of amplitude and latency for Cue P3, Target P3, SPN, and RewP to conditions of win, lose, neutral win and neutral lose; indices of amplitude for Cue P3, Target P3, SPN, and RewP to win-lose, win-neutral win, lose-neutral lose difference scores.

EFA was conducted applying promax rotation (based on correlations between ERP variables) and 15 factors (based on parallel analysis). Items were first eliminated if they loaded poorly (< 0.40 on any factor) [51, 52] and then if they loaded on more than one factor (> 0.40 on ≥ two factors) [51, 52]. Dual loading items were eliminated starting with the item whose second highest loading (absolute value) was the highest. After each elimination, parallel analysis was rerun until no additional items were indicated for removal.

Considering eigenvalues > 1 [53] and factors with > two variables [51, 52], two factors were retained (factor-item loadings ≥ 0.820). The first factor (ERPf1_TargetP3_) included Target P3 amplitude variables, to win (0.939), lose (0.919), neutral win (0.911), and neutral lose (0.912). The second factor (ERPf2_SPN_) included SPN amplitude variables to win (0.823), lose (0.899), neutral win 0.850), and neutral lose (0.820). Target P3 to win and to lose trials achieved acceptable internal consistency by the ~ tenth trial, MID SPN to win and to lose trials by the ~ 20th trial, Target P3 to neutral win and to neutral lose by the ~ 14th trial, and SPN to neutral win and to neutral lose by the ~ 26th trial (Figure S1).

*Regression analysis* Across Aim 1 and 2 models, linear regression analyses were conducted. Across Aim 1 and Aim 2 models, covariates were baseline age, sex, ADHD severity, and Depressive Problems T scores. For Aim 1 models, independent variables were ADHD PRSs; dependent variables were ERPf1_TargetP3_ and ERPf2_SPN_. For Aim 2 models, independent variables were ERPf1_TargetP3_ and ERPf2_SPN_; dependent variables were Wave 2 values of alcohol use (ESPAD binge drinking, consumption, and drunkenness subscales). For Aim 1 models, additional covariates were the first four genetic principal components and for Aim 2 models, additional covariates were baseline values of the outcome variable. *p-*values corresponding to the effect of interest were adjusted for false discovery rate (FDR) [54].

Across models, distribution of residuals was checked using normality tests (Anderson–Darling, Lilliefors-corrected Kolmogorov–Smirnov as well as visual inspection of diagnostic plots (histograms, density and Q-Q plots); homoscedasticity using the studentized Breusch-Pagan test, and multicollinearity using variance inflation factors. If the assumption of normal distribution of residuals was violated, robust linear regression analysis was conducted applying SMDM estimation with the psi function set to LQQ. Heteroscedasticity (*p*s > 0.05) and multicollinearity (VIFs < 1.24) were never observed.

To determine whether findings held accounting for behavioral performance variables, indices of reaction time (RT) to target were added as covariates to each model in sensitivity analyses conducted following identical steps as main analyses (see Supplement).

To determine whether attrition was at random, binary logistic regression analyses were conducted with age, sex, ADHD risk status, cognitive ability, socioeconomic status and Wave 1 ESPAD scores as independent variables entered simultaneously and whether an adolescent had Wave 2 data as the dependent variable.

## Results

### Attrition and basic descriptives

The model for attrition analysis was nonsignificant: χ^2^(9) = 9.266, *p* = 0.413. For descriptive statistics of and correlations across main study variables, see Tables S2 and S3.

### ERPs

**Fig. 1:**
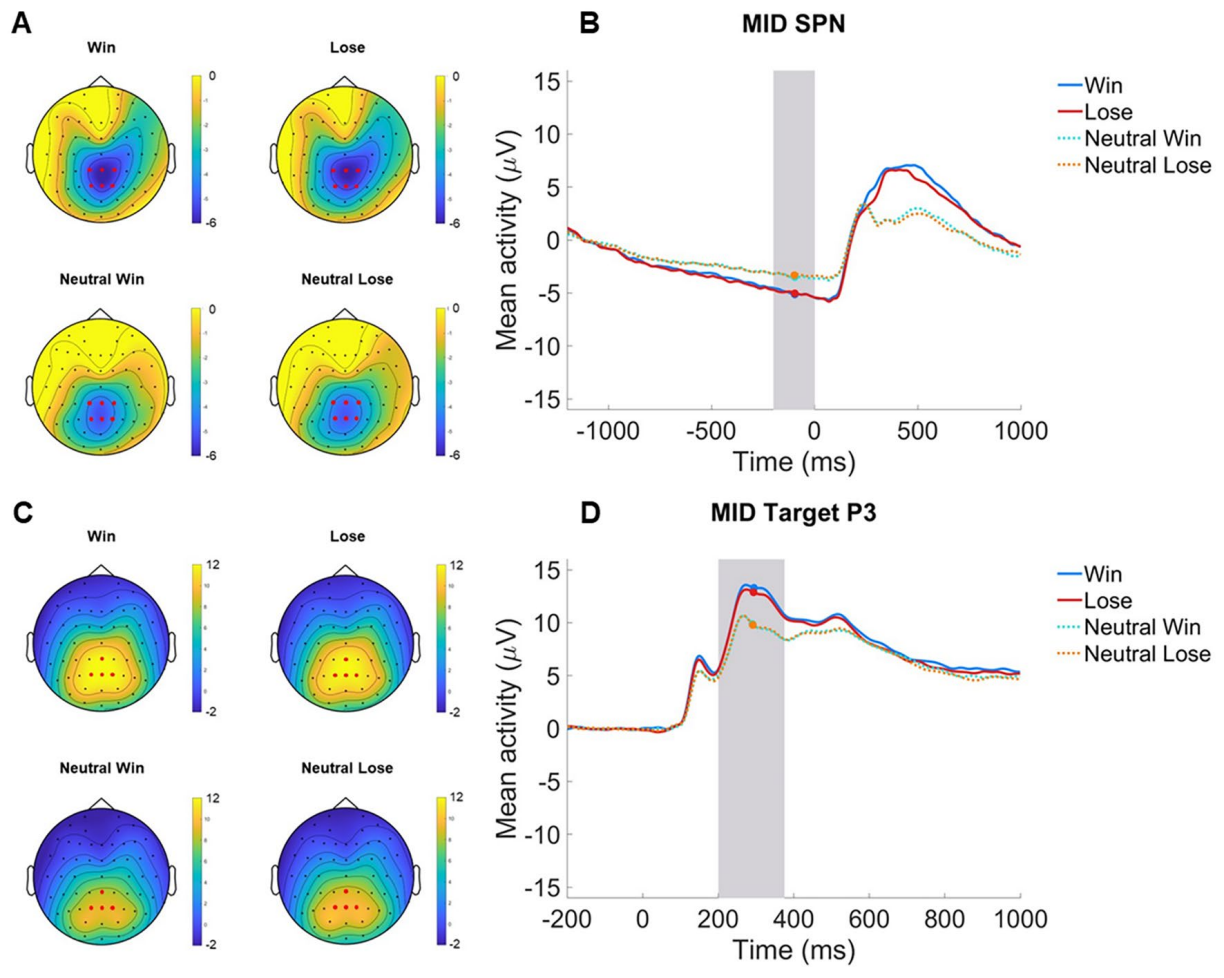
MID SPN and Target P3 in the Aim 1 analysis sample. **A** Scalp distributions depicting activation before feedback in win (SPN win), lose (SPN lose), neutral win (SPN neutral win), and neutral lose (SPN neutral lose) in the -200–0 ms time window, with electrodes selected for scoring the SPN (CPz, Pz, CP1, CP2, P1, and P2) in red. **B** ERP grand average waveforms (negative up) of the win (blue), lose (red), neutral win (cian, dotted), and neutral lose (range, dotted) condition cues. Feedback stimuli were presented at 0 ms and ERPs scored in the -200–0 ms time window indicated by grey shading. **C** Scalp distributions depicting activation to target stimuli signaling win (Target P3 win), lose (Target P3 loss), neutral win (Target P3 neutral win), and neutral lose (Target P3 neutral lose) in the 200–375 ms time window, with electrodes selected for scoring the Target P3 (CPz, Pz, P1, and P2) in red. **D** ERP grand average waveforms (negative up) of the win (blue), lose (red), neutral win (cian, dotted), and neutral lose (range, dotted) condition cues. Target stimuli were presented at 0 ms and ERPs were scored in the 200–375 ms time window indicated by grey shading. N = 282

**Fig. 2:**
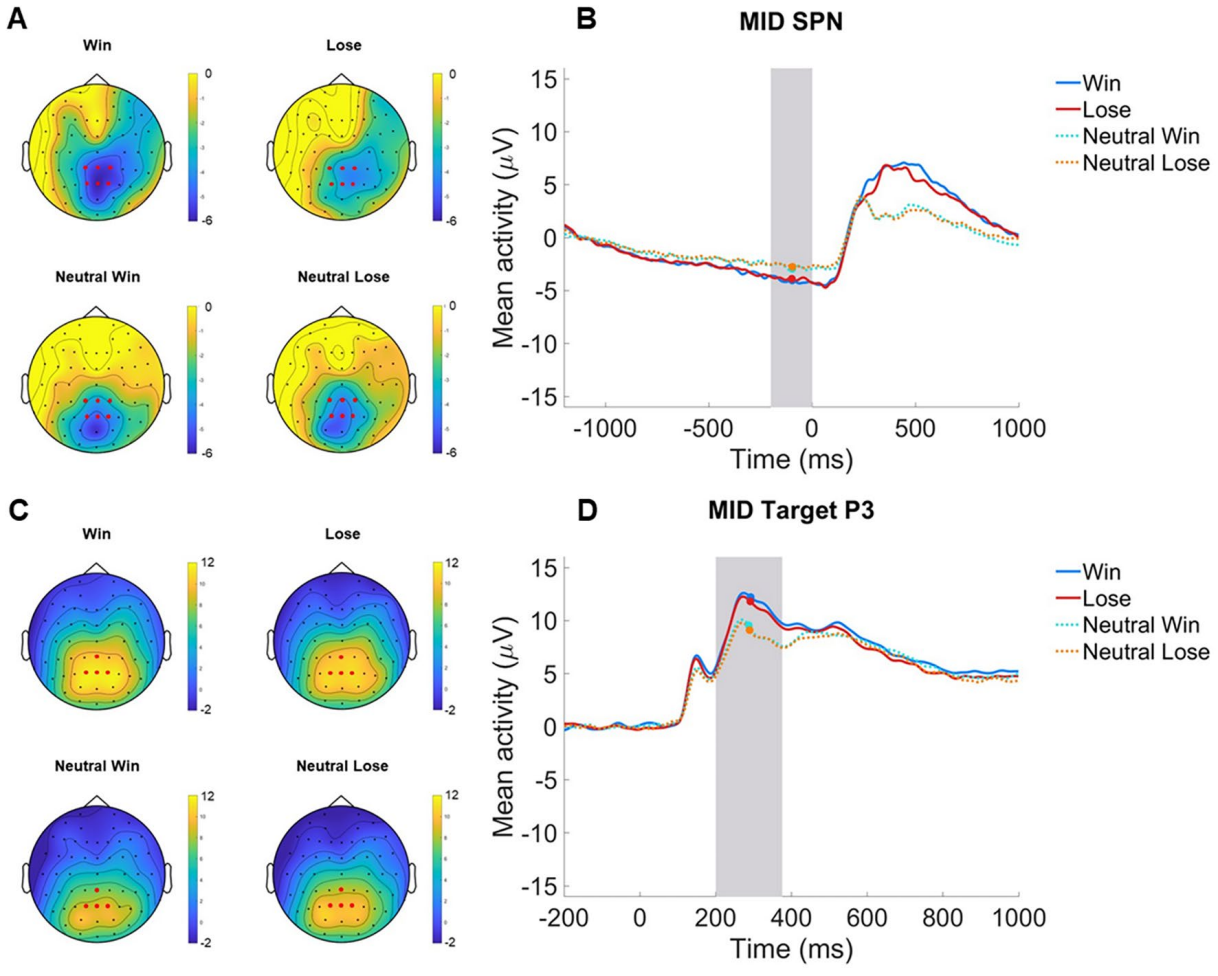
MID SPN and Target P3 in the Aim 2 analysis sample. **A** Scalp distributions depicting activation before feedback in win (SPN win), lose (SPN lose), neutral win (SPN neutral win), and neutral lose (SPN neutral lose) in the -200–0 ms time window, with electrodes selected for scoring the SPN (CPz, Pz, CP1, CP2, P1, and P2) in red. **B** ERP grand average waveforms (negative up) of the win (blue), lose (red), neutral win (cian, dotted), and neutral lose (range, dotted) condition cues. Feedback stimuli were presented at 0 ms and ERPs scored in the -200–0 ms time window indicated by grey shading. **C** Scalp distributions depicting activation to target stimuli signaling win (Target P3 win), lose (Target P3 loss), neutral win (Target P3 neutral win), and neutral lose (Target P3 neutral lose) in the 200–375 ms time window, with electrodes selected for scoring the Target P3 (CPz, Pz, P1, and P2) in red. **D** ERP grand average waveforms (negative up) of the win (blue), lose (red), neutral win (cian, dotted), and neutral lose (range, dotted) condition cues. Target stimuli were presented at 0 ms and ERPs were scored in the 200–375 ms time window indicated by grey shading. n = 98

For scalp distributions and ERP grand average waveforms for Aim 1 and 2 analyses, see Figs. 1 and 2.

### Aim 1

**Fig. 3:**
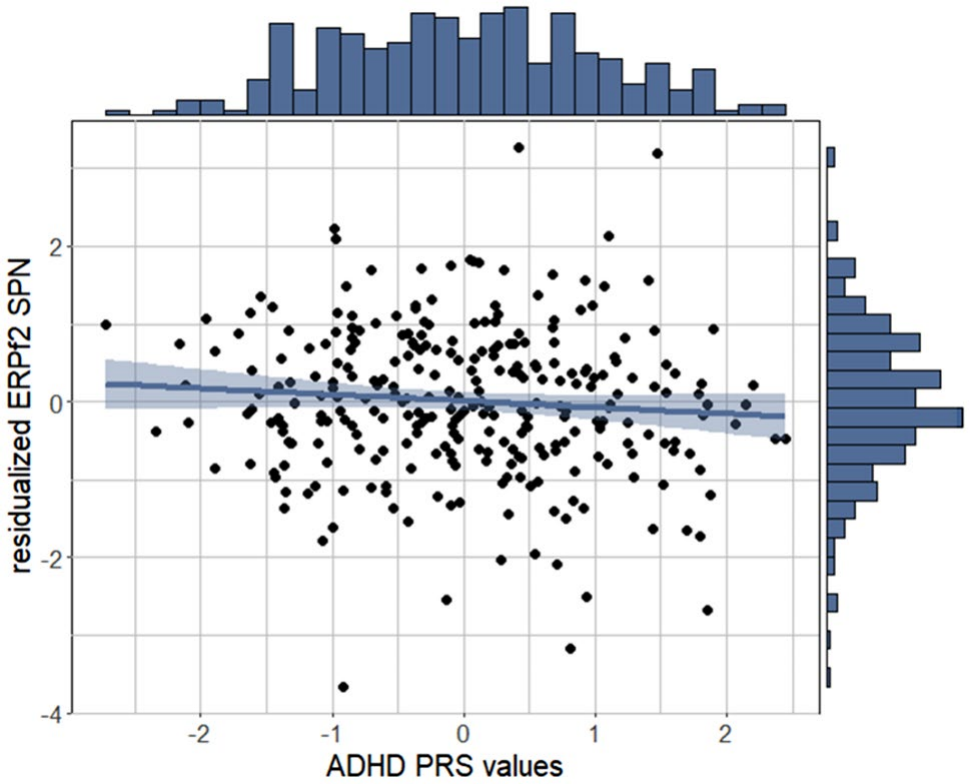
In adolescents, ADHD PRS values are associated with electrophysiological anticipation of reward. Anticipation of reward is indexed by SPN factor scores. Residualized ERPf2 SPN scores are created by regressing Genetic PC1-4, age, sex, ADHD severity, and depression scores onto standardized ADHD PRS values

The robust regression model did not predict ERPf1_TargetP3_ (χ^2^(9) = 6.552, *p* = 0.684). The linear regression model predicted ERPf2_SPN_, (*F*(9, 272) = 2.600, *p* = 0.007; adj. *R*^2^ = 0.049) (Table 1), with a negative association of standardized ADHD PRSs (*b* = -0.115, *SE* = 0.057, *p* = 0.046) and a positive association of baseline Depressive Problems scores (*b* = 0.017, *SE* = 0.007, *p* = 0.019) with ERPf2_SPN_ scores (Fig. 3). In sensitivity analyses, alternative models with behavioral performance variables as additional covariates were comparable to main models (see Supplement).

**Table 1:** Parameter estimates for linear regression model predicting ERP SPN values

	*b*	*SE*	*t*	*p*
(Intercept)	−2.363	0.867	−2.726	0.007
standardized ADHD PRSs	−0.115	0.057	−2.008	0.046
Genetic PC1	−0.392	0.998	−0.393	0.695
Genetic PC2	0.601	0.955	0.630	0.529
Genetic PC3	1.397	1.028	1.358	0.175
Genetic PC4	−0.898	0.983	−0.913	0.362
Age	0.073	0.056	1.318	0.189
Sex	0.235	0.123	1.915	0.056
ADHD severity	0.006	0.005	1.297	0.196
Depressive problems	0.017	0.007	2.363	0.019

*ADHD* attention-deficit/hyperactivity disorder, *PRS* polygenic risk score, *PC* principal component

### Aim 2

**Fig. 4:**
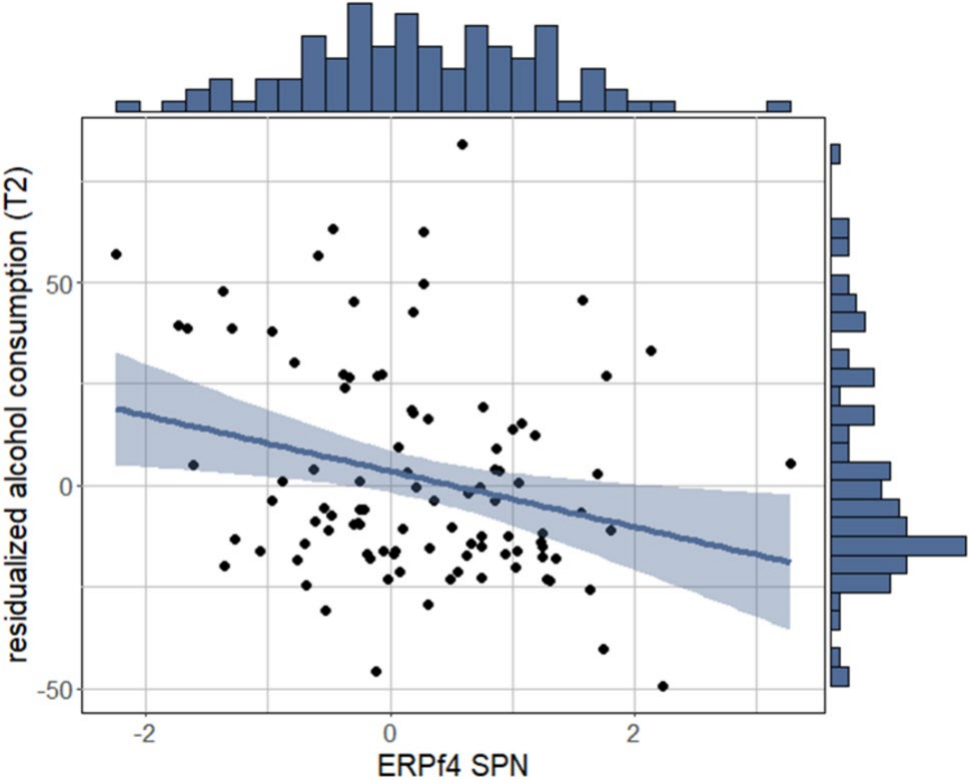
In adolescents with ADHD, electrophysiological anticipation of reward is associated with alcohol use at 18-month follow-up. Anticipation of reward is indexed by SPN factor scores. Residualized alcohol use scores are created by regressing baseline alcohol use, age, sex, ADHD severity, and depression scores onto follow-up alcohol use scores

The robust regression model with ERPf1_TargetP3_ predicted Wave 2 alcohol consumption (χ^2^(6) = 48.053, *p* < 0.001, adj. *R*^2^ = 0.295) (Table 2), with (no association of ERPf1_TargetP3_ *p*_FDR_ = 0.120, but) a positive association of baseline alcohol consumption scores (*b* = 0.814, *SE* = 0.152, *p* < 0.001) with Wave 2 alcohol consumption scores. The robust regression model with ERPf2_SPN_ predicted Wave 2 alcohol consumption (χ^2^(6) = 51.807, *p* < 0.001, adj. *R*^2^ = 0.316), with a negative association of ERPf2_SPN_ (*b* = −7.454, *SE* = 2.728, *p*_FDR_ = 0.042) and of baseline alcohol consumption scores (*b* = 0.891, *SE* = 0.146, *p* < 0.001) with Wave 2 alcohol consumption scores (Fig. 4). In sensitivity analyses, alternative models with behavioral performance variables as additional covariates were comparable to main models (see Supplement).

**Table 2:** Parameter estimates for robust linear regression model predicting alcohol use at 18-month follow-up with an effect of ERPs

	*b*	*SE*	*t*	*p*
(Intercept)	−41.222	42.806	−0.963	0.338
ERPf2_SPN_	−7.454	2.728	−2.732	0.008
Age	4.173	2.645	1.578	0.118
Sex	1.377	6.054	0.227	0.821
ADHD severity	−0.229	0.313	−0.731	0.466
Depressive problems	0.021	0.326	0.064	0.949
Alcohol consumption at baseline	0.891	0.146	6.101	< .001

*ERP* event-related potential, *ADHD* attention-deficit/hyperactivity disorder

The Wave 2 binge drinking robust regression models with ERPf1_TargetP3_ (χ^2^(6) = 48.292, *p* < 0.001, adj. *R*^2^ = 0.301) and with ERPf2_SPN_ (χ^2^(6) = 48.385, *p* < 0.001, adj. *R*^2^ = 0.301) were significant, with (no association of ERPf1_TargetP3_ *p*_FDR_ = 0.480 or ERPf2_SPN_ *p*_FDR_ = 0.480, but) a positive association of age (ERPf1_TargetP3_ model: *b* = 1.581, *SE* = 0.698, *p* = 0.026 and ERPf2_SPN_ model: *b* = 1.792, *SE* = 0.693, *p* = 0.011) and of baseline binge drinking (ERPf1_TargetP3_ model: *b* = 0.729, *SE* = 0.127, *p* < 0.001 and ERPf2_SPN_ model: *b* = 0.767, *SE* = 0.127, *p* < 0.001) with Wave 2 binge drinking scores. In sensitivity analyses, alternative models with behavioral performance variables as additional covariates were comparable to main models (see Supplement).

The Wave 2 drunkenness robust regression models with ERPf1_TargetP3_ (χ^2^(6) = 49.469, *p* < 0.001, adj. *R*^2^ = 0.444) and with ERPf2_SPN_ (χ^2^(6) = 49.261, *p* < 0.001, adj. *R*^2^ = 0.447) were significant, with (no association of ERPf1_TargetP3_ *p*_FDR_ = 0.819 or ERPf2_SPN_ *p*_FDR_ = 0.819, but) a positive association of baseline drunkenness (ERPf1_TargetP3_ model: *b* = 0.856, *SE* = 0.102, *p* < 0.001 and ERPf2_SPN_ model: *b* = 0.889, *SE* = 0.106, *p* < 0.001) with Wave 2 drunkenness scores. In sensitivity analyses, alternative models with behavioral performance variables as additional covariates were comparable to main models (see Supplement).

## Discussion

Earlier we argued that despite its advantages in clinical practice, the ADHD clinical phenotype has limitations with regard to determining etiology and explaining prognosis. We hypothesized that intermediate phenotypes may confer advantages over the clinical phenotype in these regards. To evaluate this hypothesis, we evaluated evidence for electrophysiological reward anticipation as an ADHD risk and prognostic biomarker. To this end, rather than evaluating evidence for electrophysiological reward anticipation as a biomarker of the clinical phenotype, we examined whether electrophysiological reward anticipation is associated with ADHD polygenic risk and, in individuals with the ADHD clinical phenotype, with ADHD prognosis.

Data indicate ERPs of reward anticipation and initial response to reward attainment may not be ADHD diagnostic biomarkers [7]. We have argued, however, that differentiating between groups that are defined based on clinical phenotype (i.e., with and without ADHD) by definition necessitates considerable overlap between the biomarker and the clinical phenotype. If an intermediate phenotype confers an advantage over the clinical phenotype in determining etiology and in predicting prognosis, then the intermediate phenotype will not overlap with the clinical phenotype, rather it will be associated with indices of etiology and prognosis above and beyond the clinical phenotype [7, 55].

Regarding etiology or risk, the current findings evince that ERPs of reward anticipation may be ADHD risk biomarkers insofar as ERPs of reward anticipation were associated with ADHD PRSs, above and beyond ADHD severity. These results suggest that there is an association between the biomarker and an index of ADHD etiology. Next steps for validating ERPs of reward anticipation as ADHD risk biomarkers include examining the extent to which ERPs of reward anticipation predict development of ADHD in children before the observable emergence of the clinical phenotype.

Regarding prognosis, the current findings evince that ERPs of reward anticipation may be ADHD prognostic biomarkers insofar as they are associated, longitudinally, with alcohol use as an index of ADHD prognosis, above and beyond baseline alcohol and attitude values and ADHD severity. Alcohol misuse is one of many domains of outcomes that are prognostically relevant in ADHD and current findings pertain to prognosis over 18 months. Next steps for validating ERPs of reward anticipation as prognostic biomarkers include evaluating outcomes beyond those examined here and prognosis over longer periods. Finally, to be applicable in clinical practice, neuromarkers will ultimately have to predict prognosis—based on models developed with a given group—for new individuals. The current findings reflect within-sample prospective associations and justify next steps of developing models for evaluating out-of-sample predictions.

Of note, because behavioral performance may affect the obtained results, to determine whether findings held accounting for behavioral performance variables, we conducted sensitivity analyses with RT to target added as additional covariates to each model. The findings of the original and sensitivity tests were comparable, though in case of the association between ERPs of reward anticipation and ADHD PRSs, whereas the association was significant in the original model, it minimally surpassed significance threshold (0.053) in the sensitivity model, arguably due to the addition of extra variables attenuating power.

Across aims and analyses, the SPN factor – comprising SPN amplitude to lose, to win, to neutral lose, and to neutral win and indexing anticipatory attention to, or anticipation of, informational (correct or incorrect response) and motivational (loss or win) aspects of feedback [16]—was a consistent marker of ADHD risk and prognosis. Regarding the physiological sources of the SPN, findings from spatiotemporal dipole modeling suggested the insula as a source [56]. Results from positron emission tomography (PET) [57] and functional magnetic resonance imaging (fMRI) confirmed activation in the anterior insula [58–60] and detected activation in eight additional regions [58] during paradigms probing the SPN. The anterior insula is implicated in affective-motivational processing [61], attention [62], and error-processing [63], and the additionally identified regions are implicated in anticipation (left inferior occipital gyrus and the left superior parietal lobule); arousal (right inferior parietal lobule); expectation of reward (anterior cingulate cortex, midcingulate cortex, left precentral gyrus); and processing salience (right middle frontal gyrus, bilateral insula) [58]. Greater ADHD PRS was associated with lower SPN. Lower SPN was associated with greater alcohol consumption. Accordingly, ADHD polygenic risk is associated with attenuated anticipatory attention to informational and motivational aspects of feedback and, in adolescents with the ADHD clinical phenotype, this attenuated anticipatory attention is associated with greater alcohol consumption.

Regarding ADHD risk, arguably, attenuated attention to aspects of feedback reduces the extent to which behavior is impacted and shaped by such feedback, i.e. it reduces the extent to which learning results from feedback. Consistent with this, conceptually, the ADHD clinical phenotype may be partly explained by diminished dopamine signal in anticipation of (or following) a reinforcer [64]. Empirically, ADHD in some children is associated, for example, with deficits in conditional discrimination learning [65] and perseverative responding under extinction and reversal [66]. Related to ADHD prognosis, data indicate attenuated BOLD [67] and electrophysiological [68] response to reward anticipation in individuals with alcohol dependence [67], high-risk use [68], and with a family history of alcohol dependence but low levels of use [69]. Further, attenuated BOLD response to reward anticipation predicts greater increases in alcohol use in adolescent females [70]. Finally, in the single SPN study, findings show the SPN is sensitive to craving induction in individuals with alcohol dependence [71]. Taken together, ADHD genetic risk may be associated with attenuated anticipation of aspects of feedback and this attenuated anticipation, in individuals who manifest the clinical phenotype, may confer risk for alcohol consumption.

Current findings and subsequent work may be translated into clinical practice either directly—by applying ERPs to predict ADHD prognosis or risk in clinical settings, or indirectly—by applying ERPs to inform about the biological mechanisms of behavior and impairments and through this understanding of mechanisms, to inform about targets for intervention.

Regarding direct translation, applying ERPs as predictors of ADHD risk or prognosis in clinical settings is nontrivial. Assessing ERPs is arguably more complex and time-consuming than employing certain clinical (e.g. interview, rating scale) measures. Yet, data indicate neuromarkers are concurrently and prospectively associated with education, learning, and performance as well as responses to behavioral or pharmacological treatments in children and adults; further, neuromarkers either enhance or outperform traditional measures of individual variability (e.g. educational or neuropsychological tests, interview, rating scale) [55]. By yielding unique information on individual differences in brain function and structure that influence the diversity of educational and clinical outcomes, neuromarkers appear to carry the humanitarian and practical possibility of optimizing educational and clinical practices [55]. Yet, concerns persist about availability and cost of neurophysiological measures. Validity of these concerns is questionable as the cost of a neuropsychological assessment and report often exceeds that of an fMRI [55]. Also, of neurophysiological measures, EEG is relatively cost efficient, tolerable and transportable. Finally, any economic analysis of a battery involving neurophysiological measures relative to a battery not involving those has to account for the costs associated with current practices where, e.g., children have to exhibit academic impairment before they are deemed eligible for educational treatments or where patients are prescribed treatments that may not be the most effective for them. Combination of ERPs and clinical measures may enhance clinical precision while maintaining economic efficiency and a combined approach may be clinically applicable and is promising.

Regarding indirect translation, advancing understanding of the biological correlates of ADHD risk and prognosis is informative for identifying and personalizing targets for prevention and both pharmacotherapy and psychotherapy, as deficient reinforcement learning may partly account for poor response to traditional behavioral management treatments in some youth with ADHD [72, 73].

### Limitations and strengths

We note key limitations. PRSs do not directly evince causality; PRSs are especially vulnerable to pleiotropic effects [74] and, as with all correlations, apparent associations between PRSs and ERPs may be explained by unmeasured variables or reflect indirect pathways including assortative mating, dynastic effects, or population stratification [75]. Adolescents may have underreported their alcohol use and adolescent report may be combined with objective measures or parent report in subsequent studies.

Regarding generalizability, the extent to which these findings generalize to adolescents with more severe alcohol use, to adolescents with more severe depression, or to adolescents from lower income, rural communities, is unclear. Beyond depression, other characteristics and disorders, including callous-unemotional traits and conduct disorder are also relevant for the association of ADHD with differences in reward processing [76] and these were not modeled here, but in larger samples, should be modeled.

Finally, in the current study, the “money” that was won during the task was virtual (and exchangeable for snacks as in [7, 14, 16, 34]) unlike in certain other studies where the money was given to participants as cash money at the end of the task. This difference may be also reflected in the extent to which adolescents were engaged in the task and thus magnitude of neural response to the task.

We also note strengths of the current study. We carefully characterized the sample, applied a measure of genetic risk derived from a genome-wide association study, an established and validated task to assess anticipation of reward (MID), and measures spanning different modalities (genetic, electrophysiological, rating scale). We accounted for ADHD and depression severity to ensure findings are not driven by overlap between ADHD PRSs and ADHD severity or are explainable by depression.

## Conclusion

Converging evidence across models indicates electrophysiological indices of anticipation of reward are associated, principally, with ADHD genetic risk and prognosis, but not depression severity. Specifically, amplitude values of ERPs reflecting anticipatory attention to, or anticipation of, informational (correct or incorrect response) and motivational (loss or win) aspects of feedback are associated, in adolescents, with ADHD PRSs and in adolescents at-risk for ADHD, with alcohol use. These electrophysiological indices of anticipation of reward may thus be biomarkers of ADHD risk and prognosis.

## Supplementary Information

Below is the link to the electronic supplementary material.

Supplementary file 1

## Data Availability

Datasets generated for this study are available at: https://osf.io/2p9hc/

## References

[1] American Psychiatric Association (2022) Diagnostic and statistical manual of mental disorders, fifth edition, text revision (DSM-5-TR). Washington, D.C, American Psychiatric Association

[2] Luderer M, Ramos Quiroga JA, Faraone SV, Zhang James Y, Reif A (2021) Alcohol use disorders and ADHD. Neurosci Biobehav Rev. 10.1016/j.neubiorev.2021.07.010 34265320 10.1016/j.neubiorev.2021.07.010

[3] Nigg JT, Sibley MH, Thapar A, Karalunas SL (2020) Development of ADHD: etiology, heterogeneity, and early life course. Annu Rev Dev Psychol 2:559–583. 10.1146/annurev-devpsych-060320-093413 34368774 10.1146/annurev-devpsych-060320-093413 PMC8336725

[4] Nigg JT, Karalunas SL, Feczko E, Fair DA (2020) Toward a revised nosology for attention-deficit/hyperactivity disorder heterogeneity. Biolog Psychiatry Cogn Neurosci Neuroimag. 10.1016/j.bpsc.2020.02.005 10.1016/j.bpsc.2020.02.005 PMC7423612 32305325

[5] Nigg JT, Karalunas SL, Gustafsson HC, Bhatt P, Ryabinin P, Mooney MA et al (2020) Evaluating chronic emotional dysregulation and irritability in relation to ADHD and depression genetic risk in children with ADHD. J Child Psychol Psychiatry 61:205–214. 10.1111/jcpp.13132 31605387 10.1111/jcpp.13132 PMC6980250

[6] Fair DA, Bathula D, Nikolas MA, Nigg JT, Iyer S, Bathula D et al (2012) Distinct neuropsychological subgroups in typically developing youth inform heterogeneity in children with ADHD. Proc Natl Acad Sci 6:80. 10.1073/pnas.1115365109 10.1073/pnas.1115365109 PMC3340031 22474392

[7] Hámori G, File B, Fiáth R, Pászthy B, Réthelyi JM, Ulbert I et al (2023) Adolescent ADHD and electrophysiological reward responsiveness: a machine learning approach to evaluate classification accuracy and prognosis. Psychiatry Res 323:115139. 10.1016/j.psychres.2023.115139 36921508 10.1016/j.psychres.2023.115139

[8] Umeda-Yano S, Fujimoto M (2015) Intermediate phenotype approach for neuropsychiatric disorders. Neurodegen Dis Syst Dis. 10.1007/978-4-431-54541-5_7

[9] Rasetti R, Weinberger DR (2011) Intermediate phenotypes in psychiatric disorders. Curr Opin Genet Dev. 10.1016/j.gde.2011.02.003 21376566 10.1016/j.gde.2011.02.003 PMC3138621

[10] Califf RM (2018) Biomarker definitions and their applications. Exp Biol Med 243:213–221. 10.1177/1535370217750088 10.1177/1535370217750088 PMC5813875 29405771

[11] Groen Y, Gaastra GF, Lewis-Evans B, Tucha O (2013) Risky behavior in gambling tasks in individuals with adhd—a systematic literature review. PLoS ONE 8:e74909. 10.1371/journal.pone.0074909 24058638 10.1371/journal.pone.0074909 PMC3772864

[12] Sjöwall D, Roth L, Lindqvist S, Thorell LB (2013) Multiple deficits in ADHD: Executive dysfunction, delay aversion, reaction time variability, and emotional deficits. J Child Psychol Psychiatry 54:619–627. 10.1111/jcpp.12006 23061803 10.1111/jcpp.12006 PMC3758957

[13] Solanto MV, Gilbert SN, Raj A, Zhu J, Pope-Boyd S, Stepak B et al (2007) Neurocognitive functioning in AD/HD, predominantly inattentive and combined subtypes. J Abnorm Child Psychol 35:729–744. 10.1007/s10802-007-9123-6 17629724 10.1007/s10802-007-9123-6 PMC2265203

[14] Rádosi A, Ágrez K, Pászthy B, Réthelyi JM, Ulbert I, Bunford N (2023) Concurrent and prospective associations of reward response with affective and alcohol problems: ADHD-related differential vulnerability. J Youth Adolesc. 10.1007/s10964-023-01794-7 37270465 10.1007/s10964-023-01794-7

[15] Hajcak G, Weinberg A, Macnamara A, Foti D (2011) ERPs and the study of Emotionpdf. In: Stephen JL (ed) Handbook of event-related potential components. Oxford University Press, New York, NY, pp 441–472

[16] Hámori G, Rádosi A, Pászthy B, Réthelyi JM, Ulbert I, Fiáth R et al (2022) Reliability of reward ERPs in middle-late adolescents using a custom and a standardized preprocessing pipeline. Psychophysiology. 10.1111/psyp.14043 35298041 10.1111/psyp.14043 PMC9541384

[17] Jeste SS, Frohlich J, Loo SK (2015) Electrophysiological biomarkers of diagnosis and outcome in neurodevelopmental disorders. Curr Opin Neurol 28:110–116. 10.1097/WCO.0000000000000181 25710286 10.1097/WCO.0000000000000181 PMC7334029

[18] Michelini G, Norman LJ, Shaw P, Loo SK (2022) Treatment biomarkers for ADHD: taking stock and moving forward. Transl Psychiatry 12:1–30. 10.1038/s41398-022-02207-2 36224169 10.1038/s41398-022-02207-2 PMC9556670

[19] Chronaki G, Benikos N, Soltesz F, Sonuga-Barke EJS (2019) The reinforcing value of delay escape in attention deficit/hyperactivity disorder: an electrophysiological study Clinical. Neuroimage. 10.1016/j.nicl.2019.101917 10.1016/j.nicl.2019.101917 PMC6614592 31491823

[20] Holroyd CB, Baker TE, Kerns KA, Müller U, Muller U (2008) Electrophysiological evidence of atypical motivation and reward processing in children with attention-deficit hyperactivity disorder. Neuropsychologia 46:2234–2242. 10.1016/j.neuropsychologia.2008.02.011 18367216 10.1016/j.neuropsychologia.2008.02.011

[21] Kohls G, Herpertz-Dahlmann B, Konrad K (2009) Hyperresponsiveness to social rewards in children and adolescents with attention-deficit/hyperactivity disorder (ADHD). Behav Brain Funct 5:20 19426488 10.1186/1744-9081-5-20 PMC2685404

[22] Wiersema JR, van der Meere JJ, Roeyers H (2005) ERP correlates of impaired error monitoring in children with ADHD. J Neural Transm (Vienna) 112:1417–1430. 10.1007/s00702-005-0276-6 15726277 10.1007/s00702-005-0276-6

[23] Wiersema JR, van der Meere JJ, Roeyers H (2009) ERP correlates of error monitoring in adult ADHD. J Neural Transm (Vienna) 116:371–379. 10.1007/s00702-008-0165-x 19093191 10.1007/s00702-008-0165-x

[24] Wild-Wall N, Oades RD, Schmidt-Wessels M, Christiansen H, Falkenstein M (2009) Neural activity associated with executive functions in adolescents with attention-deficit/hyperactivity disorder (ADHD). Int J Psychophysiol 74:19–27. 10.1016/j.ijpsycho.2009.06.003 19607863 10.1016/j.ijpsycho.2009.06.003

[25] Thoma P, Edel M-A, Suchan B, Bellebaum C (2015) Probabilistic reward learning in adults with attention deficit hyperactivity disorder-an electrophysiological study. Psychiatry Res 225:133–144. 10.1016/j.psychres.2014.11.006 25467706 10.1016/j.psychres.2014.11.006

[26] Rosch KS, Hawk LW (2013) The effects of performance-based rewards on neurophysiological correlates of stimulus, error, and feedback processing in children with ADHD. Psychophysiology 50:1157–1173. 10.1111/psyp.12127 24033316 10.1111/psyp.12127 PMC3807761

[27] Bunford N, Kujawa A, Dyson M, Olino T, Klein DN (2022) Examination of developmental pathways from preschool temperament to early adolescent ADHD symptoms through initial responsiveness to reward. Dev Psychopathol 34:841–853. 10.1017/S0954579420002199 33722319 10.1017/S0954579420002199

[28] Kujawa A, Hajcak G, Klein DN (2019) Reduced reward responsiveness moderates the effect of maternal depression on depressive symptoms in offspring: evidence across levels of analysis. J Child Psychol Psychiatry. 10.1111/jcpp.12944 29978904 10.1111/jcpp.12944 PMC6296896

[29] Wechsler D. (2003) Wechsler intelligence scale for children–Fourth Edition (WISC-IV). 10.1080/09297040590951587 16306021

[30] Wechsler D. (2008) Wechsler adult intelligence scale–Fourth Edition (WAIS–IV).

[31] Stoet G (2017) PsyToolkit: a novel web-based method for running online questionnaires and reaction-time experiments. Teach Psychol 44:24–31. 10.1177/0098628316677643

[32] Stoet G (2010) PsyToolkit: a software package for programming psychological experiments using Linux. Behav Res Methods 42:1096–1104. 10.3758/BRM.42.4.1096 21139177 10.3758/BRM.42.4.1096

[33] DuPaul GJ, Power TJ, Anastopoulos AD, Reid R (2016) ADHD rating scale-5 for children and adolescents. The Guilford Press, New York - London

[34] Rádosi A, Pászthy B, Welker T, Zubovics EA, Réthelyi JM, Ulbert I et al (2021) The association between reinforcement sensitivity and substance use is mediated by individual differences in dispositional affectivity in adolescents. Addict Behav 114:106719. 10.1016/j.addbeh.2020.106719 33160749 10.1016/j.addbeh.2020.106719

[35] Sebők-Welker T, Posta E, Ágrez K, Rádosi A, Zubovics E, Réthelyi JM et al (2023) The association between prenatal maternal stress and adolescent affective outcomes is mediated by childhood maltreatment and adolescent behavioral inhibition system sensitivity. Child Psychiatry Hum Dev. 10.1007/s10578-023-01499-9 36738426 10.1007/s10578-023-01499-9 PMC11362206

[36] Nárai Á, Hermann P, Rádosi A, Vakli P, Weiss B, Réthelyi JM et al (2024) Amygdala volume is associated with ADHD risk and severity beyond comorbidities in adolescents: clinical testing of brain chart reference standards. Res Child Adoles Psychopathol. 10.1007/s10802-024-01190-0 10.1007/s10802-024-01190-0 PMC11217056 38483760

[37] Központi Statisztikai Hivatal. A háztartások életszínvonala, 2020. GYORSTÁJÉKOZTATÓ Keresetek, 2021 Március 2021. https://www.ksh.hu/docs/hun/xftp/idoszaki/hazteletszinv/2020/index.html. Accessed 24 Oct 2024

[38] Kraus L, Nociar A. 2016 ESPAD report 2015: results from the European school survey project on alcohol and other drugs. Luxembourg: European Monitoring Centre for Drugs and Drug Addiction.

[39] Elekes Z. 2012 ESPAD 2011 (Európai Iskolavizsgálat a fiatalok alkohol- és egyéb drogfogyasztási szokásairól) ötödik hullámának magyarországi adatfelvétele. Budapest, Hungary.

[40] Molinaro S, Siciliano V, Curzio O, Denoth F, Mariani F (2012) Concordance and consistency of answers to the self-delivered ESPAD questionnaire on use of psychoactive substances. Int J Methods Psychiatr Res. 10.1002/mpr.1353 22359402 10.1002/mpr.1353 PMC6878571

[41] Hibell B, Guttormsson U, Ahlström S, Balakireva O, Bjarnason T, Kokkevi A, et al. 2012. The 2011 ESPAD Report: Substance Use Among Students in 36 European Countries.

[42] Knutson B, Fong GW, Adams CM, Varner JL, Hommer D (2001) Dissociation of reward anticipation and outcome with event-related fMRI. NeuroReport 12:3683–3687. 10.1097/00001756-200112040-00016 11726774 10.1097/00001756-200112040-00016

[43] Knutson B, Fong GW, Bennett SM, Adams CM, Hommer D (2003) A region of mesial prefrontal cortex tracks monetarily rewarding outcomes: characterization with rapid event-related fMRI. Neuroimage 18:263–272. 10.1016/S1053-8119(02)00057-5 12595181 10.1016/s1053-8119(02)00057-5

[44] NIMH. 2011. Positive Valence Systems: Workshop Proceedings.

[45] Broyd SJ, Richards HJ, Helps SK, Chronaki G, Bamford S, Sonuga-Barke EJ (2012) An electrophysiological monetary incentive delay (e-MID) task: a way to decompose the different components of neural response to positive and negative monetary reinforcement. J Neurosci Methods 209(1):40–49. 10.1016/j.jneumeth.2012.05.015 22659003 10.1016/j.jneumeth.2012.05.015

[46] Zubovics EA, Fiáth R, Rádosi A, Pászthy B, Réthelyi JM, Ulbert I et al (2021) Neural and self-reported reward responsiveness are associated with dispositional affectivity and emotion dysregulation in adolescents with evidence for convergent and incremental validity. Psychophysiology 58:e13723. 10.1111/psyp.13723 33179791 10.1111/psyp.13723

[47] Chronaki G, Soltesz F, Benikos N, Sonuga-Barke EJS (2017) An electrophysiological investigation of reinforcement effects in attention deficit/hyperactivity disorder: dissociating cue sensitivity from down-stream effects on target engagement and performance. Dev Cogn Neurosci 28:12–20. 10.1016/j.dcn.2017.10.003 29080475 10.1016/j.dcn.2017.10.003 PMC6987869

[48] Illumina, Inc. Infinium HTS Assay Reference Guide (Document # 15045738 v04) 2019.

[49] Lall R, Robinson T (2022) The MIDAS touch: accurate and scalable missing-data imputation with deep learning. Polit Anal 30:179–196. 10.1017/pan.2020.49

[50] Demontis D, Walters GB, Athanasiadis G, Walters R, Therrien K, Nielsen TT et al (2023) Genome-wide analyses of ADHD identify 27 risk loci, refine the genetic architecture and implicate several cognitive domains. Nat Genet 55:198–208. 10.1038/s41588-022-01285-8 36702997 10.1038/s41588-022-01285-8 PMC10914347

[51] Watkins M (2021) A step-by-step guide to exploratory factor analysis with SPSS, 1st edn. Routledge, New York, NY

[52] Tabachnick BG, Fidell LS (2021) Using multivariate statistics, 7th edn. Allyn & Bacon/Pearson Education, Boston, MA

[53] Costello A, Osborne J (2019) Best practices in exploratory factor analysis: four recommendations for getting the most from your analysis. Pract Assess Res Eval. 10.7275/jyj1-4868

[54] Benjamini Y, Hochberg Y (1995) Controlling the false discovery rate: a practical and powerful approach to multiple testing. J Roy Stat Soc: Ser B (Methodol) 57:289–300. 10.1111/j.2517-6161.1995.tb02031.x

[55] Gabrieli JDE, Ghosh SS, Whitfield-Gabrieli S (2015) Prediction as a humanitarian and pragmatic contribution from human cognitive neuroscience. Neuron 85:11–26. 10.1016/j.neuron.2014.10.047 25569345 10.1016/j.neuron.2014.10.047 PMC4287988

[56] Böcker KBE, Brunia CHM, van den Berg-Lenssen MMC (1994) A spatiotemporal dipole model of the stimulus preceding negativity (spn) prior to feedback stimuli. Brain Topogr. 10.1007/BF01184839 7803202 10.1007/BF01184839

[57] Brunia CHM, De Jong BM, Van Den Berg-Lenssen MMC, Paans AMJ (2000) Visual feedback about time estimation is related to a right hemisphere activation measured by PET. Exp Brain Res. 10.1007/s002219900293 10706432 10.1007/s002219900293

[58] Kotani Y, Ohgami Y, Ishiwata T, Arai J, Kiryu S, Inoue Y (2015) Source analysis of stimulus-preceding negativity constrained by functional magnetic resonance imaging. Biol Psychol 111:53–64. 10.1016/j.biopsycho.2015.08.005 26307468 10.1016/j.biopsycho.2015.08.005

[59] Tsukamoto T, Kotani Y, Ohgami Y, Omura K, Inoue Y, Aihara Y (2006) Activation of insular cortex and subcortical regions related to feedback stimuli in a time estimation task: an fMRI study. Neurosci Lett 399:39–44. 10.1016/j.neulet.2006.01.061 16490307 10.1016/j.neulet.2006.01.061

[60] Kotani Y, Ohgami Y, Kuramoto Y, Tsukamoto T, Inoue Y, Aihara Y (2009) The role of the right anterior insular cortex in the right hemisphere preponderance of stimulus-preceding negativity (SPN): An fMRI study. Neurosci Lett. 10.1016/j.neulet.2008.11.032 19028549 10.1016/j.neulet.2008.11.032

[61] Craig AD (2003) Interoception: the sense of the physiological condition of the body. Curr Opin Neurobiol 13:500–505. 10.1016/s0959-4388(03)00090-4 12965300 10.1016/s0959-4388(03)00090-4

[62] Nelson SM, Dosenbach NUF, Cohen AL, Wheeler ME, Schlaggar BL, Petersen SE (2010) Role of the anterior insula in task-level control and focal attention. Brain Struct Funct 214:669–680. 10.1007/s00429-010-0260-2 20512372 10.1007/s00429-010-0260-2 PMC2886908

[63] Czobor P, Kakuszi B, Németh K, Balogh L, Papp S, Tombor L et al (2017) Electrophysiological indices of aberrant error-processing in adults with ADHD: a new region of interest. Brain Imaging Behav 11:1616–1628. 10.1007/s11682-016-9610-x 27752922 10.1007/s11682-016-9610-x

[64] Sagvolden T, Johansen EB, Aase H, Russell VA (2005) A dynamic developmental theory of attention-deficit/hyperactivity disorder (ADHD) predominantly hyperactive/impulsive and combined subtypes. Behav Brain Sci 28:397–419. 10.1017/S0140525X05000075 16209748 10.1017/S0140525X05000075

[65] De Meyer H, Beckers T, Tripp G, van der Oord S (2019) Deficits in conditional discrimination learning in children with ADHD are independent of delay aversion and working memory. J Clin Med 8:1381. 10.3390/jcm8091381 31484457 10.3390/jcm8091381 PMC6780856

[66] Itami S, Uno H (2002) Orbitofrontal cortex dysfunction in attention-deficit hyperactivity disorder revealed by reversal and extinction tasks. NeuroReport. 10.1097/00001756-200212200-00016 12499848 10.1097/00001756-200212200-00016

[67] Zeng J, You L, Yang F, Luo Y, Yu S, Yan J et al (2023) A meta-analysis of the neural substrates of monetary reward anticipation and outcome in alcohol use disorder. Hum Brain Mapp 44:2841–2861. 10.1002/hbm.26249 36852619 10.1002/hbm.26249 PMC10089105

[68] Komarnyckyj M, Retzler C, Cao Z, Ganis G, Murphy A, Whelan R et al (2022) At-risk alcohol users have disrupted valence discrimination during reward anticipation. Addict Biol 27:e13174. 10.1111/adb.13174 35470555 10.1111/adb.13174 PMC9286798

[69] Yau W-YW, Zubieta J-K, Weiland BJ, Samudra PG, Zucker RA, Heitzeg MM (2012) Nucleus accumbens response to incentive stimuli anticipation in children of alcoholics: relationships with precursive behavioral risk and lifetime alcohol use. J Neurosci 32:2544–2551. 10.1523/JNEUROSCI.1390-11.2012 22396427 10.1523/JNEUROSCI.1390-11.2012 PMC3567451

[70] Swartz JR, Weissman DG, Ferrer E, Beard SJ, Fassbender C, Robins RW et al (2020) Reward-related brain activity prospectively predicts increases in alcohol use in adolescents. J Am Acad Child Adolesc Psychiatry 59:391–400. 10.1016/j.jaac.2019.05.022 31173884 10.1016/j.jaac.2019.05.022 PMC6891148

[71] Sehrig S, Odenwald M, Rockstroh B (2020) Feedback-related brain potentials indicate the influence of craving on decision-making in patients with alcohol use disorder: an experimental study. Eur Addict Res 27:216–226. 10.1159/000511417 33291101 10.1159/000511417

[72] Evans SW, Owens JS, Bunford N (2014) Evidence-based psychosocial treatments for children and adolescents evidence-based psychosocial treatments for children and adolescents with disruptive behavior. J Clin Child Adolesc Psychol 43:527–551. 10.1080/15374410701820117 24245813 10.1080/15374416.2013.850700 PMC4025987

[73] Evans SW, Owens JS, Wymbs BT, Ray AR (2018) Evidence-based psychosocial treatments for children and adolescents with attention deficit/hyperactivity disorder. J Clin Child Adolesc Psychol 47:157–198 29257898 10.1080/15374416.2017.1390757

[74] Martin AR, Daly MJ, Robinson EB, Hyman SE, Neale BM (2019) Predicting polygenic risk of psychiatric disorders. Biol Psychiatry 86:97–109. 10.1016/j.biopsych.2018.12.015 30737014 10.1016/j.biopsych.2018.12.015 PMC6599546

[75] Chen C, Lu Y, Lundström S, Larsson H, Lichtenstein P, Pettersson E (2022) Associations between psychiatric polygenic risk scores and general and specific psychopathology symptoms in childhood and adolescence between and within dizygotic twin pairs. J Child Psychol Psychiatry 63:1513–1522. 10.1111/jcpp.13605 35292971 10.1111/jcpp.13605 PMC9790278

[76] Hawes SW, Waller R, Byrd AL, Bjork JM, Dick AS, Sutherland MT et al (2021) Reward processing in children with disruptive behavior disorders and callous-unemotional Traits in the ABCD Study. Am J Psychiatry 178:333–342. 10.1176/appi.ajp.2020.19101092 32731811 10.1176/appi.ajp.2020.19101092 PMC7855017

