# Supplementary material for: Electrophysiological indices of reward anticipation as ADHD risk and prognostic biomarkers
Source: Eur Child Adolesc Psychiatry. 2024 Nov 8;34(6):1905–16. doi: 10.1007/s00787-024-02606-4 (PMC12198071; doi:10.1007/s00787-024-02606-4)

**SUPPLEMENTARY INFORMATION TO:**

**ELECTROPHYSIOLOGICAL INDICES OF REWARD ANTICIPATION**

**AS ADHD PROGNOSTIC AND RISK BIOMARKERS**

**Supplementary Methods**

**Participants**

Medication washout information is reported for visits/ timepoints from which obtained data were used in current analyses. At baseline, of 132 adolescents at-risk for ADHD, *n*=74 (56.1%) were medication-naїve; of those currently using ADHD medication (*n*_stimulants_=10 (47.6%), *n*_nonstimulants_=5 (23.8%)), for the first assessment session, 14 took a ≥24-hour medication washout prior to testing and one did not and for the second assessment session, 10 took a ≥24-hour medication washout prior to testing, one did not, and three did not indicate whether or not they took a washout. At Wave 2, of 99 adolescents at-risk for ADHD, *n*=74 (74.7%) were medication-naїve; of those currently using ADHD medication (*n*_stimulants_=1 (5.6%), *n*_nonstimulants_=17 (94.4%)), for the first assessment session, 11 took a ≥24-hour medication washout prior to testing, four did not, and three did not indicate whether or not they took a washout.

**Measures**

***Adolescent self-report measures***

***Alcohol use.*** Alcohol use was measured using the European School Survey Project on Alcohol and Other Drugs (ESPAD) master questionnaire [1], a self-report measure of alcohol, cigarette, and other drug use, energy drink use, gaming, and internet use. For the current study, nine items were used to measure the lifetime, the past 12-month, and the past 30-day presence and severity of alcohol consumption (3 items), binge drinking (3 items), and drunkenness (3 items). Alcohol consumption is assessed based on the question, “*On how many occasions (if any) have you had any alcoholic beverage to drink?*”. Adolescents rate this question as applied to (1) their lifetime, (2) during the last 12 months, and (3) during the last 30 days, on a scale ranging from 0 to ≥40, with higher scores indicating more alcohol consumption. Binge drinking is assessed based on the question, “*How many times (if any) have you had five or more drinks on one occasion?*”. Adolescents rate this question as applied to (1) their lifetime, (2) during the last 12 months, and (3) during the last 30 days, on a scale ranging from 0 to ≥10, with higher scores indicating more binge drinking. Drunkenness is assessed based on the question, “*On how many occasions (if any) have you been intoxicated from drinking alcoholic beverages, for example staggered when walking, not being able to speak properly, throwing up or not remembering what happened?*”. Adolescents rate this question as applied to (1) their lifetime, (2) during the last 12 months, and (3) during the last 30 days, on a scale ranging from 0 to ≥40, with higher scores indicating more drunkenness.

Prior findings indicate both the original (e.g., internal consistency, test-retest reliability [2,3] and cross-cultural comparability [1]) and the Hungarian translation (construct validity [4,5]) have acceptable psychometric properties. In the current study, consumption subscale (sum of 3 consumption items), binge drinking subscale (sum of 3 binge drinking items), and drunkenness subscale (sum of 3 drunkenness items) were used to index alcohol consumption, binge drinking, and drunkenness. In the current sample, the binge drinking (ω_baseline_=.940; ω_T2_=.938), the consumption (ω_baseline_=.916; ω_T2_=.934), and the drunkenness (ω_baseline_=.944; ω_T2_=.938) subscales exhibited acceptable internal consistency and were used in analyses.

***Parent-report measures***

***ADHD.*** ADHD was measured using the ADHD Rating Scale-5 (ARS 5) [6], is a 30-item parent- and teacher-report measure of the past 6-month presence and severity of DSM-5 ADHD symptoms across two domains and impairment (inattention symptoms (9 items), e.g. “*Fails to give close attention to details or makes careless mistakes in schoolwork, at work, or during other activities*” and hyperactivity/impulsivity symptom (9 items), e.g. “*Fidgets with or taps hands or feet or squirms in seat*”) and functional impairment across six domains: relationship with significant others (family members for the home version), e.g. “*Getting along with family members*”, relationship with peers, e.g. “*Getting along with other teenagers*”, academic functioning, e.g. “*Performing academically in school*”, behavioral functioning “*Controlling behavior in school*”, homework performance, e.g. “*Completing or returning homework*”, and self-esteem, e.g. “*Feeling good about himself/herself*” (2×6 impairment items, with one set corresponding to inattention and one to hyperactivity/impulsivity). Parents and teachers rate items on a four-point scale ranging in case of symptoms from 0 (never or rarely) to 4 (very often) and in case of impairment from 0 (no problem) to 3 (severe problem), with higher scores indicating more severe symptoms and impairment. Items make up two symptoms subscales, Inattention and Hyperactivity-Impulsivity, and a Total scale.

The ARS-5 has a child (5-10 years) and an adolescent form (11-17 years) form – with age-appropriate and DSM-5 compatible descriptions of symptoms; and a home (parent-report) and a school (teacher-report) version. In the current study, the adolescent home (i.e., parent-report) version was used. Prior findings indicate both the original (e.g., internal consistency and 6-week test-retest reliability; factor structure; concurrent validity and predictive validity [6]) and the Hungarian translation (internal consistency [7,8]) have acceptable psychometric properties. In the current sample, the ARS-5 Total exhibited acceptable internal consistency (ω_baseline_=.954) and was used in analyses.

***Experimental paradigm and procedures***

First, adolescents were familiarized with the EEG laboratory and procedures. Second, they were fitted with electrodes and seated in a chair in the testing room (a darkened Faraday shield) approximately 50 cm away from the monitor. Third, adolescents participated in an electrophysiological Monetary Incentive Delay (eMID) task [9,10], to probe reward anticipation. Adolescents were asked to sit as still as possible during recording.

In the eMID task, adolescents are presented with three types of stimuli: cue, target, and feedback. *Cue* stimuli inform participants about trial type: gain, loss, or control. In gain trials, indicated by a crossed circle, participants can win (+1000 HUF); in loss trials, indicated by a crossed square, participants can avoid losing (-1000 HUF); and in neutral trials (empty circle or square, i.e. a neutral gain and a neutral loss condition), there is no monetary consequence. The association between circle and square-with the outcomes they represent (winning or losing) was counterbalanced across participants. Following each cue (2000 ms duration), there is an anticipatory phase (duration between 2000 and 2500 ms), during which participants wait for and are briefly presented with a target stimulus that they have to respond to as quickly as they can by pressing the number 7 on an external numeric keypad to win or avoid losing money. Participants were instructed to respond to neutral stimuli the same way. Success or failure was indicated above a fixation cross on the computer screen (monetary outcome of the current trial) (2000 ms feedback duration), and the accumulated money in total was indicated below the fixation cross. The duration of the intertrial interval was between 1000 and 2000 ms.

First, a practice block of 24 trials is presented. During the practice block, participants’ reaction time is measured and is used to calibrate the experimental blocks such that participants are set to win 66% of the time. Then, five experimental blocks of 240 trials (12 in each block / condition) are presented with trial types presented with equal probability and in random order. To maximize effectiveness of the experimental manipulation, participants were told that the virtual money they accumulated can be exchanged for snacks (candy, chips, etc.) they chose before the beginning of recording.

***PRS***

**Quality Control (QC).** The initial dataset contained *N*=333 individuals and 745,980 variants; the genotyping call rate was 98.73%. Before QC, copy number variants, insertions-deletions, pseudo-autosomal and non-autosomal regions were removed. *n*=12 participants were also excluded to ensure only a single sibling was present from each family. QC was conducted with PLINK (versions 1.90b6.26 and 1.90b7 [11]) and custom scripts [12].

Following Marees et al. [13], QC steps included exclusion of (1/i) variants with missing rate >20%, (1/ii) individuals with genotype missing rate >20%, (1/iii) variants with missing rate >1%, (1/iv) individuals with genotype missing rate >1%; (2) individuals with sex discrepancy (i.e., a discrepancy between genotype-based vs. self-reported sex information); (3) variants with minor allele frequency <1%; (4) variants deviating from the Hardy-Weinberg equilibrium at *p*<1e-6; (5) individuals with a heterozygosity rate exceeding the sample mean by ±3 *SD*s after variant pruning (window size 1500kb, step size 150 variants, pairwise *r*^2^ threshold .2); and (6) cryptically related individuals (pi-hat>.1875). In total, 307 participants and 177060 pruned variants were carried forward to imputation.

To assess the potential population substructure, the first 20 genomic principal components were extracted with principal component analysis. Four principal components were retained based on a Scree plot (explaining 5.28%, 5.20%, 5.11% and 5.01% variance, respectively). Pairwise scatterplots were examined between these 4 principal components and indicated no apparent population stratification but identified a handful of potential outliers.

DNA strands were aligned to the 1000 Genomes Project (1kGP) Phase 3 reference dataset [14] by using Genotype Harmonizer (v1.4.25) [15]. We used the default parameters, except for not excluding the unreferenced variants from the dataset.

**Imputation.** Files were converted to the Oxford GEN format with fcGENE (v1.0.7) [16] and phased with SHAPEIT2 (v2.r904) [16,17] by using the following parameters: 7 burn-in, 8 pruning and 20 main Markov Chain Monte Carlo iterations; 100 conditioning states in ~2.0Mb windows with an effective population size of 15000. Phased data were imputed with IMPUTE2 (v2.3.2) [18,19] in ~5Mb windows with 250kb buffer on each side using 500 hidden Markov model states and an effective population size of 20000. The 1kGP Phase 3 dataset was used as the reference panel during both phasing and imputation. Imputed variants with an INFO score <.8 or best-guess genotype certainty (*r*^2^) <.9 were excluded. After imputation, 7145485 variants were present in our dataset. Post-imputation QC included removal of duplicated variants and positions [12], leaving 7105467 variants for further analysis.

**PRS Calculation**. PRS values were calculated with PRSice-2 (v2.3.5) [20] based on a discovery dataset involving 38691 individuals with ADHD and 186843 controls [21]. Default settings were applied with appropriate case and control numbers. Effective and non-effective alleles were checked on the base data. As alleles were fixed previously, .02% of variants were incorrectly positioned. PRS values were calculated for a binary trait (ADHD diagnosis) using default settings, with 79 individuals with ADHD and 208 controls. Of the 6774224 variants present in the discovery set, 3572964 were included for further processing. After clumping, 131673 variants remained and were used for PRS calculation. *p*-value thresholds were assessed for the inclusion of variants at intervals of 5e-05 between *p*=5e-08 and *p*=.50, as well as at *p*=1. Using SNP cutoff of *p*<.50, the number of ADHD PRS SNPs was 99330 with an associated *R*^2^ of ≈3.7%. Raw PRSs were standardized before statistical analysis.

**Supplementary Results**

**Sensitivity analyses**

Because behavioral performance variables were highly correlated (*r*s≥.78), sensitivity testing was conducted with mean reaction time to gain and SD of the mean reaction time to gain entered as additional covariates.

**Sensitivity analyses to Aim 1 with behavioral variables**

The robust regression model predicted ERPf1_TargetP3_ (χ^2^(11)=42.778, *p*<.001, adj. *R*^2^=.101), with (no association of standardized ADHD PRSs with ERPf1_TargetP3_ *p*=.562, but) a negative association of RT to win (*b*=-9.278, *SE*=2.722, *p*<.001) and of baseline Depressive Problems scores (*b*=-.015, *SE*=.007, *p*=.045) with ERPf1_TargetP3._ The regression model predicted ERPf2_SPN_ (*F*(11, 269)=2.469, *p*=.006, adj. *R*^2^=.055) with (no association of standardized ADHD PRSs with ERPf2_SPN_ *p*=.054, but) a positive association of sex (*b*=.249, *SE*=.124, *p*=.046) and of baseline Depressive Problems scores (*b*=.018, *SE*=.007, *p*=.015) with ERPf2_SPN_.

**Sensitvitiy analyses to Aim 2 with behavioral variables**

The robust regression model with ERPf1_TargetP3_ predicted Wave 2 alcohol consumption (χ^2^(8)=49.292, *p*<.001, adj. *R*^2^=.282), with (no association of ERPf1_TargetP3_ *p*=.164, but) a positive association of baseline alcohol consumption scores (*b*=.810, *SE*=.154, *p*<.001) with Wave 2 alcohol consumption scores. The robust regression model with ERPf2_SPN_ predicted Wave 2 alcohol consumption (χ^2^(8)=58.381, *p*<.001, adj. *R*^2^=.327), with a negative association of ERPf2_SPN_ (*b*=-7.694, *SE*=2.707, *p*=.006) and a positive association of baseline alcohol consumption scores (*b*=.865, *SE*=.146, *p*<.001) with Wave 2 alcohol consumption scores (Table S4).

The Wave 2 binge drinking robust regression models with ERPf1_TargetP3_ (χ^2^(8)=50.511, *p*<.001, adj. *R*^2^=.296) and with ERPf2_SPN_ (χ^2^(8)=53.696, *p*<.001, adj. *R*^2^=.312) were significant, with (no association of ERPf1_TargetP3_ *p*=.640 or ERPf2_SPN_ *p*=.111, but) a positive association of age (ERPf1_TargetP3_ model: *b*=1.640, *SE*=.714, *p*=.024 and ERPf2_SPN_ model: *b*=1.743, *SE*=.698, *p*=.014) and of baseline binge drinking (ERPf1_TargetP3_ model: *b*=.731, *SE*=.129, *p*<.001 and ERPf2_SPN_ model: *b*=.750, *SE*=.128, *p*<.001) with Wave 2 binge drinking scores.

The Wave 2 drunkenness robust regression models with ERPf1_TargetP3_ (χ^2^(8)=53.901, *p*<.001, adj. *R*^2^=.440) and with ERPf2_SPN_ (χ^2^(8)=53.420, *p*<.001, adj. *R*^2^=.447) were significant, with (no association of ERPf1_TargetP3_ *p*=.444 or ERPf2_SPN_ *p*_FDR_=.371, but) a positive association of baseline drunkenness (ERPf1_TargetP3_ model: *b*=.867, *SE*=.106, *p*<.001 and ERPf2_SPN_ model: *b*=.903, *SE*=.109, *p*<.001) with Wave 2 drunkenness scores.

**Supplementary References**

[1] Kraus L, Nociar A. ESPAD report 2015: results from the European school survey project on alcohol and other drugs. Luxembourg: European Monitoring Centre for Drugs and Drug Addiction; 2016.

[2] Hibell B, Guttormsson U, Ahlström S, Balakireva O, Bjarnason T, Kokkevi A, et al. The 2011 ESPAD Report: Substance Use Among Students in 36 European Countries. 2012. https://doi.org/ISBN: 978-91-7278-233-4.

[3] Molinaro S, Siciliano V, Curzio O, Denoth F, Mariani F. Concordance and consistency of answers to the self-delivered ESPAD questionnaire on use of psychoactive substances: ESPAD reliability. Int J Methods Psychiatr Res 2012;21:158–68. https://doi.org/10.1002/mpr.1353.

[4] Elekes Z. ESPAD 2011 (Európai Iskolavizsgálat a fiatalok alkohol- és egyéb drogfogyasztási szokásairól) ötödik hullámának magyarországi adatfelvétele. Budapest, Hungary: 2012.

[5] Rádosi A, Pászthy B, Welker T, Zubovics EA, Réthelyi JM, Ulbert I, et al. The association between reinforcement sensitivity and substance use is mediated by individual differences in dispositional affectivity in adolescents. Addict Behav 2021;114:106719. https://doi.org/10.1016/j.addbeh.2020.106719.

[6] DuPaul GJ, Power TJ, Anastopoulos AD, Reid R. ADHD Rating Scale-5 for Children and Adolescents. New York - London: The Guilford Press; 2016.

[7] Hámori G, File B, Fiáth R, Pászthy B, Réthelyi JM, Ulbert I, et al. Adolescent ADHD and electrophysiological reward responsiveness: A machine learning approach to evaluate classification accuracy and prognosis. Psychiatry Res 2023;323:115139. https://doi.org/10.1016/j.psychres.2023.115139.

[8] Rádosi A, Ágrez K, Pászthy B, Réthelyi JM, Ulbert I, Bunford N. Concurrent and Prospective Associations of Reward Response with Affective and Alcohol Problems: ADHD-Related Differential Vulnerability. J Youth Adolesc 2023;52:1856–72. https://doi.org/10.1007/s10964-023-01794-7.

[9] Knutson B, Fong GW, Adams CM, Varner JL, Hommer D. Dissociation of reward anticipation and outcome with event-related fMRI: Neuroreport 2001;12:3683–7. https://doi.org/10.1097/00001756-200112040-00016.

[10] Knutson B, Fong GW, Bennett SM, Adams CM, Hommer D. A region of mesial prefrontal cortex tracks monetarily rewarding outcomes: characterization with rapid event-related fMRI. NeuroImage 2003;18:263–72. https://doi.org/10.1016/S1053-8119(02)00057-5.

[11] Chang CC, Chow CC, Tellier LC, Vattikuti S, Purcell SM, Lee JJ. Second-generation PLINK: rising to the challenge of larger and richer datasets. GigaScience 2015;4:s13742-015-0047–8. https://doi.org/10.1186/s13742-015-0047-8.

[12] Coleman JRI, Euesden J, Patel H, Folarin AA, Newhouse S, Breen G. Quality control, imputation and analysis of genome-wide genotyping data from the Illumina HumanCoreExome microarray. Brief Funct Genomics 2016;15:298–304. https://doi.org/10.1093/bfgp/elv037.

[13] Marees AT, de Kluiver H, Stringer S, Vorspan F, Curis E, Marie-Claire C, et al. A tutorial on conducting genome-wide association studies: Quality control and statistical analysis. Int J Methods Psychiatr Res 2018;27:e1608. https://doi.org/10.1002/mpr.1608.

[14] Auton A, Abecasis GR, Altshuler DM, Durbin RM, Abecasis GR, Bentley DR, et al. A global reference for human genetic variation. Nature 2015;526:68–74. https://doi.org/10.1038/nature15393.

[15] Deelen P, Bonder MJ, van der Velde KJ, Westra H-J, Winder E, Hendriksen D, et al. Genotype harmonizer: automatic strand alignment and format conversion for genotype data integration. BMC Res Notes 2014;7:901. https://doi.org/10.1186/1756-0500-7-901.

[16] Roshyara NR, Scholz M. fcGENE: A Versatile Tool for Processing and Transforming SNP Datasets. PLOS ONE 2014;9:e97589. https://doi.org/10.1371/journal.pone.0097589.

[17] Delaneau O, Marchini J, Zagury J-F. A linear complexity phasing method for thousands of genomes. Nat Methods 2012;9:179–81. https://doi.org/10.1038/nmeth.1785.

[18] Howie BN, Donnelly P, Marchini J. A Flexible and Accurate Genotype Imputation Method for the Next Generation of Genome-Wide Association Studies. PLOS Genet 2009;5:e1000529. https://doi.org/10.1371/journal.pgen.1000529.

[19] Howie B, Marchini J, Stephens M. Genotype Imputation with Thousands of Genomes. G3 GenesGenomesGenetics 2011;1:457–70. https://doi.org/10.1534/g3.111.001198.

[20] Choi SW, O’Reilly PF. PRSice-2: Polygenic Risk Score software for biobank-scale data. GigaScience 2019;8:giz082. https://doi.org/10.1093/gigascience/giz082.

[21] Demontis D, Walters GB, Athanasiadis G, Walters R, Therrien K, Nielsen TT, et al. Genome-wide analyses of ADHD identify 27 risk loci, refine the genetic architecture and implicate several cognitive domains. Nat Genet 2023;55:198–208. https://doi.org/10.1038/s41588-022-01285-8.

[22] Zeileis A, Hothorn T. Diagnostic Checking in Regression Relationships. R News 2002;2:7–10.

[23] Steiner M, Grieder S, Revelle W, Auerswald M, Moshagen M, Ruscio J, et al. EFAtools: An R package with fast and flexible implementations of exploratory factor analysis tools. J Open Source Softw 2020;5:2521. https://doi.org/10.21105/joss.02521.

[24] Fox J, Weisberg S. An R Companion to Applied Regression. 3rd ed. Thousand Oaks, CA: Sage; 2019.

[25] Lall R, Robinson T. The MIDAS Touch: Accurate and Scalable Missing-Data Imputation with Deep Learning. Polit Anal 2022;30:179–96. https://doi.org/10.1017/pan.2020.49.

[26] Lall R, Robinson T. Efficient Multiple Imputation for Diverse Data in Python and R: MIDASpy and rMIDAS. J Stat Softw 2023;107:1–38. https://doi.org/10.18637/jss.v107.i09.

[27] Gross J, Ligges U. Five omnibus tests for testing the composite hypothesis of normality 2015.

[28] Maechler M, Rousseeuw P, Croux C, Todorov V, Ruckstuhl A, Salibian-Barrera M, et al. robustbase: Basic Robust Statistics. R package 2023.

[29] Koller M, Stahel WA. Sharpening Wald-type inference in robust regression for small samples. Comput Stat Data Anal 2011;55:2504–15. https://doi.org/10.1016/j.csda.2011.02.014.

[30] Broyd SJ, Richards HJ, Helps SK, Chronaki G, Bamford S, Sonuga-Barke EJS. An electrophysiological monetary incentive delay (e-MID) task: A way to decompose the different components of neural response to positive and negative monetary reinforcement. J Neurosci Methods 2012;209:40–9. https://doi.org/10.1016/j.jneumeth.2012.05.015.

| Table S1 | | | |
| --- | --- | --- | --- |
| *R packages employed for analyses.* | | | |
| statistical method/ test | R package | version number | reference |
| ADHD PRS calculation | PRSice-2 | v2.3.5 | [20] |
| Breusch-Pagan test | lmtest | v0.9-40 | [22] |
| Exploratory factor analysis | EFAtools | v0.4.4 | [23] |
| multicollinearity | car | v3.1-2 | [24] |
| multiple imputation | rMIDAS | v1.0.0 | [25,26] |
| normality tests | nortest | v1.0-4 | [27] |
| robust linear regression analysis | robustbase | v0.99-1 | [28,29] |

| Table S2  *Descriptive statistics of main study variables*. | | | | | | |
| --- | --- | --- | --- | --- | --- | --- |
|  | *n* | *M* | *SD* | Median | Min | Max |
| ADHD severity | 304 | 16.43 | 12.75 | 14.00 | 0 | 52 |
| ADHD PRSs (standardized) | 287 | 0 | 1 | -.04 | -2.72 | 2.45 |
| ERPf1_TargetP3_ | 297 | 0 | .96 | -.09 | -2.21 | 4.16 |
| ERPf2_SPN_ | 297 | .01 | .97 | -.02 | -3.66 | 3.27 |
| binge drinking (T1) | 304 | 3.16 | 5.91 | 0 | 0 | 24 |
| consumption (T1) | 304 | 15.93 | 21.26 | 6.50 | 0 | 95 |
| drunkenness (T1) | 304 | 3.66 | 9.11 | 0 | 0 | 74 |
| binge drinking (T2) | 232 | 7.02 | 8.12 | 3 | 0 | 30 |
| consumption (T2) | 232 | 32.59 | 29.48 | 24 | 0 | 110 |
| drunkenness (T2) | 232 | 9.31 | 15.84 | 3 | 0 | 84 |
| depression (T1) | 304 | 56.22 | 8.54 | 53 | 50 | 92 |
| *Notes*. T1=wave 1 (baseline); T2=wave 2 (18-month follow-up); binge drinking=ESPAD binge drinking; consumption=ESPAD consumption; drunkenness=ESPAD drunkenness; depression=YSR Depressive Problems T-score. | | | | | | |

| Table S3  *Rank correlations between main study variables*. | | | | | | | | | | | |
| --- | --- | --- | --- | --- | --- | --- | --- | --- | --- | --- | --- |
|  |  | 1. | 2. | 3. | 4. | 5. | 6. | 7. | 8. | 9. | 10. |
| 1. ADHD severity | τb (*p*) | - | - | - | - | - | - | - | - | - | - |
|  | *p* | - | - | - | - | - | - | - | - | - | - |
|  | 95% CI | - | - | - | - | - | - | - | - | - | - |
| 2. ADHD PRSs (standardized) | τb | .098 | - | - | - | - | - | - | - | - | - |
|  | *p* | .096 | - | - | - | - | - | - | - | - | - |
|  | 95% CI | -.017; .212 | - | - | - | - | - | - | - | - | - |
| 3. ERPf1_TargetP3_ | τb | -.044 | .047 | - | - | - | - | - | - | - | - |
|  | *p* | .453 | .430 | - | - | - | - | - | - | - | - |
|  | 95% CI | -.157; .070 | -.070; .163 | - | - | - | - | - | - | - | - |
| 4. ERPf2_SPN_ | τb | .080 | -.057 | -.305 | - | - | - | - | - | - | - |
|  | *p* | .168 | .340 | <.001 | - | - | - | - | - | - | - |
|  | 95% CI | -.034; .192 | -.173; .060 | -.405; -.199 | - | - | - | - | - | - | - |
| 5. binge drinking (T1) | τb | -.071 | -.017 | .045 | .023 | - | - | - | - | - | - |
|  | *p* | .220 | .772 | .443 | .697 | - | - | - | - | - | - |
|  | 95% CI | -.182; .042 | -.133; .099 | -.069; .158 | -.091; .136 | - | - | - | - | - | - |
| 6. consumption (T1) | τb | -.071 | .004 | .021 | .041 | .584 | - | - | - | - | - |
|  | *p* | .220 | .941 | .712 | .480 | <.001 | - | - | - | - | - |
|  | 95% CI | -.182; .042 | -.111; .120 | -.093; .135 | -.073; .154 | .504; .653 | - | - | - | - | - |
| 7. drunkenness (T1) | τb | -.011 | -.012 | .030 | .033 | .643 | .559 | - | - | - | - |
|  | *p* | .851 | .839 | .610 | .572 | <.001 | <.001 | - | - | - | - |
|  | 95% CI | -.123; .102 | -.128; .104 | -.084; .143 | -.081; .146 | .571; .704 | .476; .631 | - | - | - | - |
| 8. binge drinking (T2) | τb | -.017 | -.004 | -.022 | .065 | .440 | .422 | .398 | - | - | - |
|  | *p* | .795 | .956 | .737 | .324 | <.001 | <.001 | <.001 | - | - | - |
|  | 95% CI | -.146; .112 | -.136; .128 | -.151; .107 | -.064; .192 | .330; .538 | .310; .523 | .284; .501 | - | - | - |
| 9. consumption (T2) | τb | -.043 | .048 | .033 | -.010 | .388 | .485 | .393 | .586 | - | - |
|  | *p* | .510 | .478 | .621 | .885 | <.001 | <.001 | <.001 | <.001 | - | - |
|  | 95% CI | -.171; .086 | -.085; .179 | -.097; .161 | -.138; .119 | .273; .492 | .380; .578 | .278; .496 | .495; .665 | - | - |
| 10. drunkenness (T2) | τb | -.011 | .018 | -.056 | .037 | .361 | .376 | .481 | .624 | .544 | - |
|  | *p* | .865 | .786 | .400 | .573 | <.001 | <.001 | <.001 | <.001 | <.001 | - |
|  | 95% CI | -.140; .118 | -.114; .150 | -.183; .074 | -.092; .165 | .243; .468 | .260; .481 | .375; .574 | .539; .697 | .446; .628 | - |
| 11. depression (T1) | τb | .112 | .035 | -.060 | .054 | .154 | .160 | .171 | .054 | .104 | .124 |
|  | *p* | .050 | .559 | .306 | .354 | .007 | .005 | .003 | .416 | .113 | .059 |
|  | 95% CI | .000; .222 | -.081; .150 | -.172; .055 | -.060; .167 | .042; .262 | .048; .267 | .060; .278 | -.076; .181 | -.025; .230 | -.005; .249 |
| *Notes*. τb=Kendall’s tau-b rank correlation coefficient; T1=wave 1 (baseline); T2=wave 2 (18-month follow-up); binge drinking=ESPAD binge drinking; consumption=ESPAD consumption; drunkenness=ESPAD drunkenness; depression=YSR Depressive Problems T-score. | | | | | | | | | | | |

| Table S4 | | | | |
| --- | --- | --- | --- | --- |
| *Parameter estimates for robust linear regression model in sensitivity analyses predicting alcohol use at 18-month follow-up with an effect of ERPs.* | | | | |
|  | *b* | *SE* | *t* | *p* |
| (Intercept) | -13.995 | 47.700 | -.293 | .770 |
| *M* RT to gain* | -45.812 | 108.266 | -.423 | .673 |
| *SD* RT to gain* | -79.785 | 176.586 | -.452 | .652 |
| ERPf2_SPN_ | -7.694 | 2.707 | -2.843 | .006 |
| Age | 3.888 | 2.637 | 1.474 | .144 |
| Sex | .360 | 6.054 | .059 | .953 |
| ADHD severity | -.235 | .320 | -.733 | .466 |
| Depressive problems | -.060 | .328 | -.184 | .854 |
| Alcohol consumption at baseline | .865 | .146 | 5.932 | <.001 |
| *Note*. ERP=event-related potential; ADHD=attention-deficit/hyperactivity disorder; RT=reaction time.  *: RT data were trimmed to remove responses faster than 150 milliseconds and exceeded ±2.5 SD around the mean response time as in [30]. | | | | |


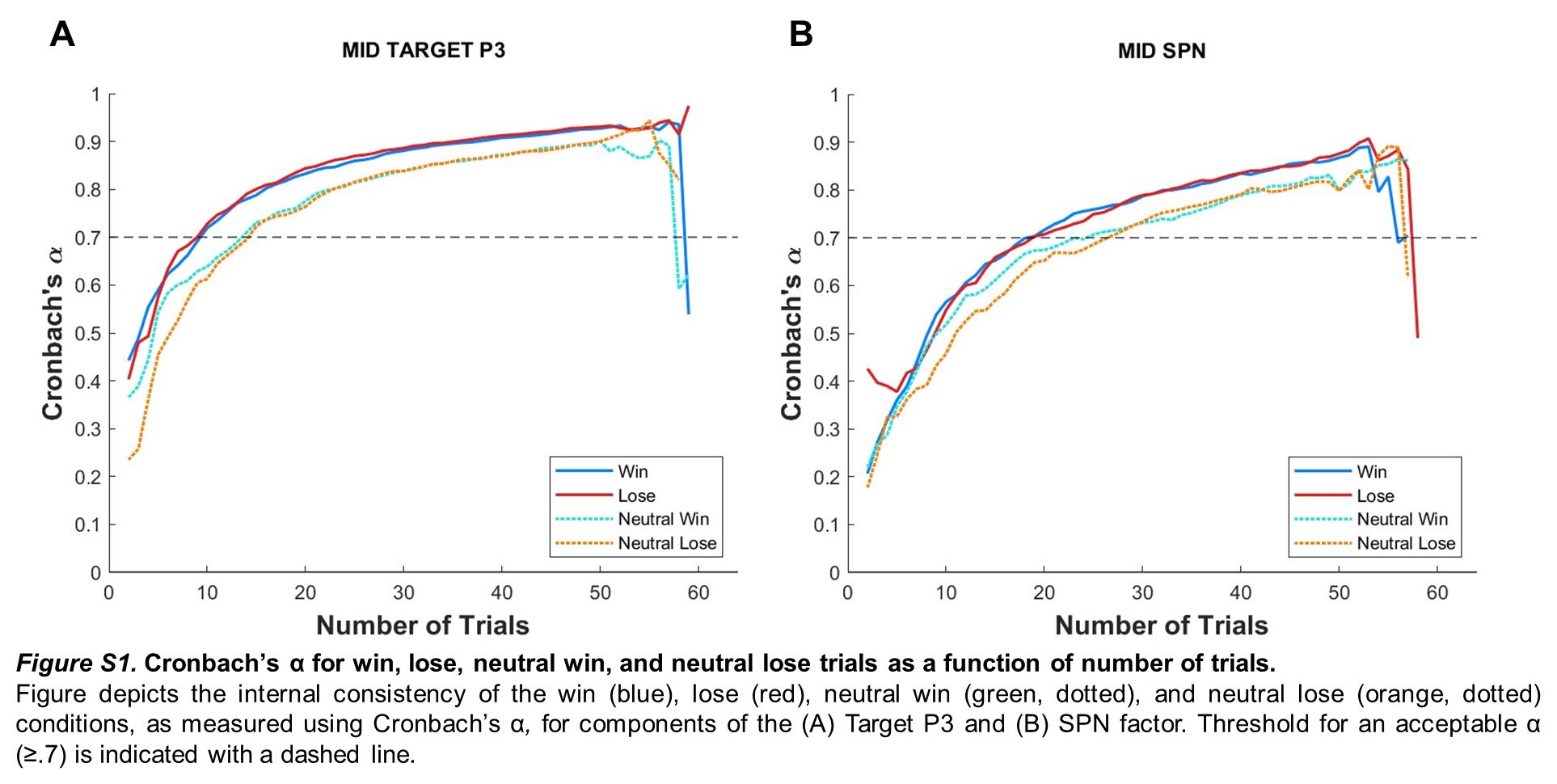

Supplement: Supplementary file 1 [file 787_2024_2606_MOESM1_ESM.docx]
